# Supplementary figures and images for: KAT6B is required for histone 3 lysine 9 acetylation and SOX gene expression in the developing brain
Source: Life Sci Alliance. 2024 Nov 13;8(2):e202402969. doi: 10.26508/lsa.202402969 (PMC11561263; doi:10.26508/lsa.202402969)

Figure 1 H3K9ac, H3K23ac and panH3

NSPCs H3K9ac

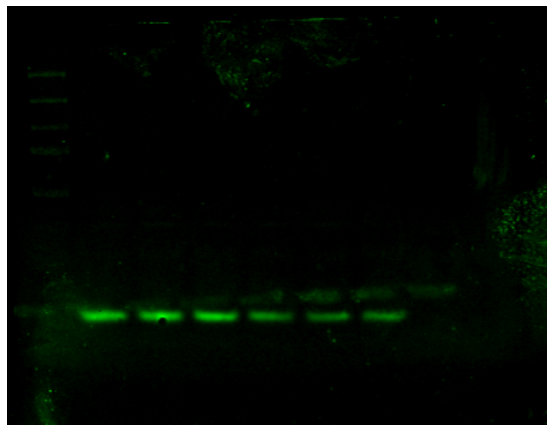

NSPCs H3K23ac

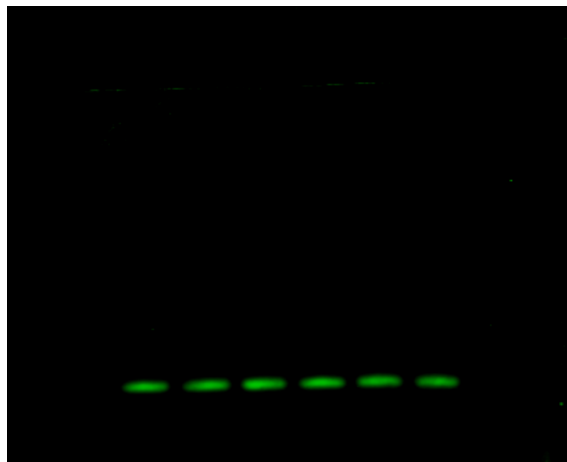

E12.5 dTel H3K9ac

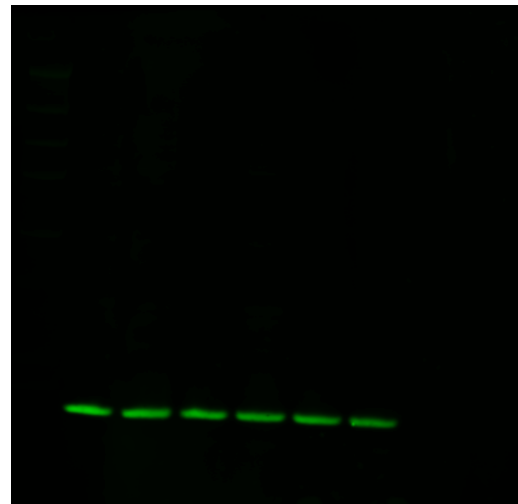

E12.5 dTel H3K23ac

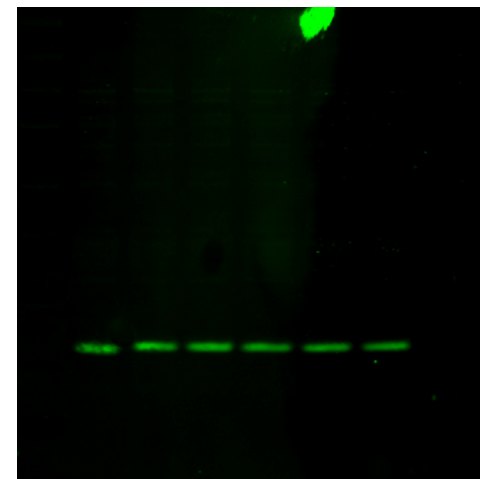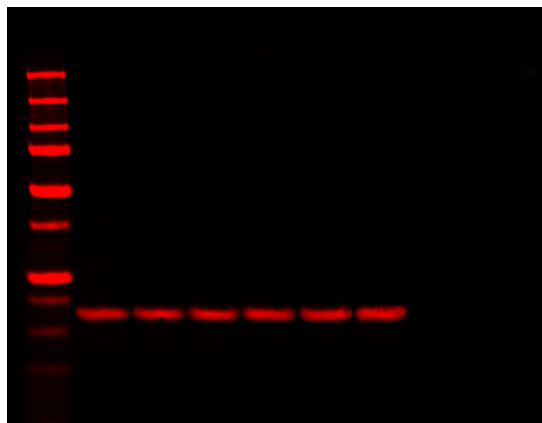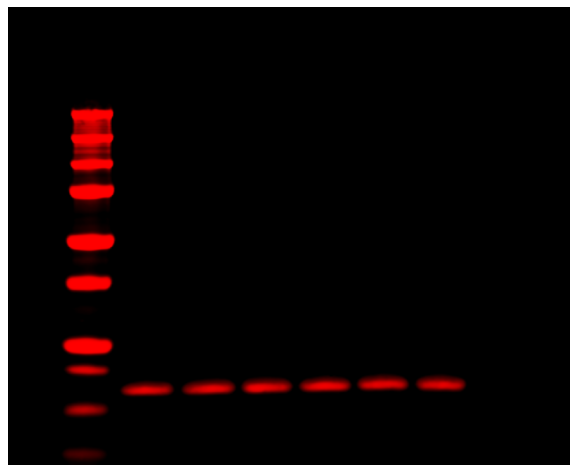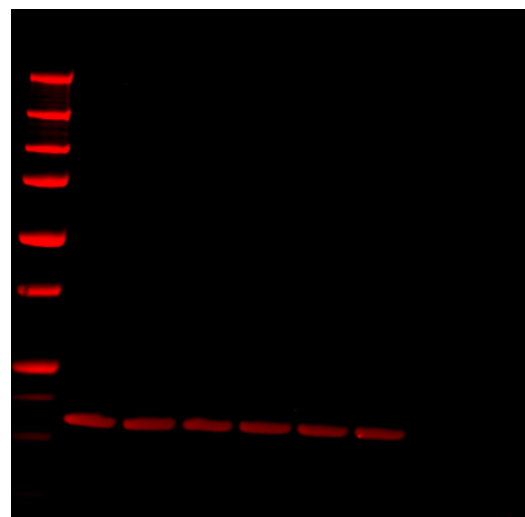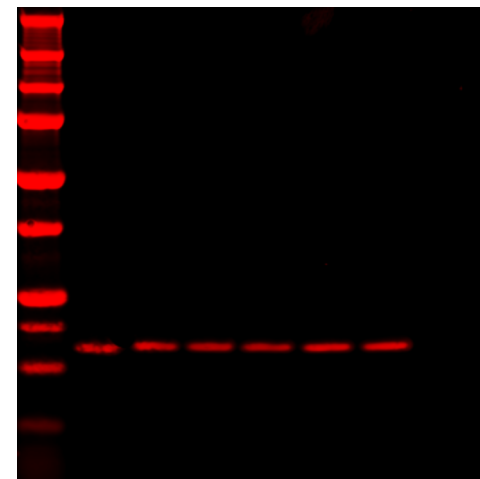

Supplement: Supplementary file 1 [file LSA-2024-02969_SdataF1.1.pdf]

Supplemental Figure 2 Kat6b<sup>-/-</sup> vs. WT

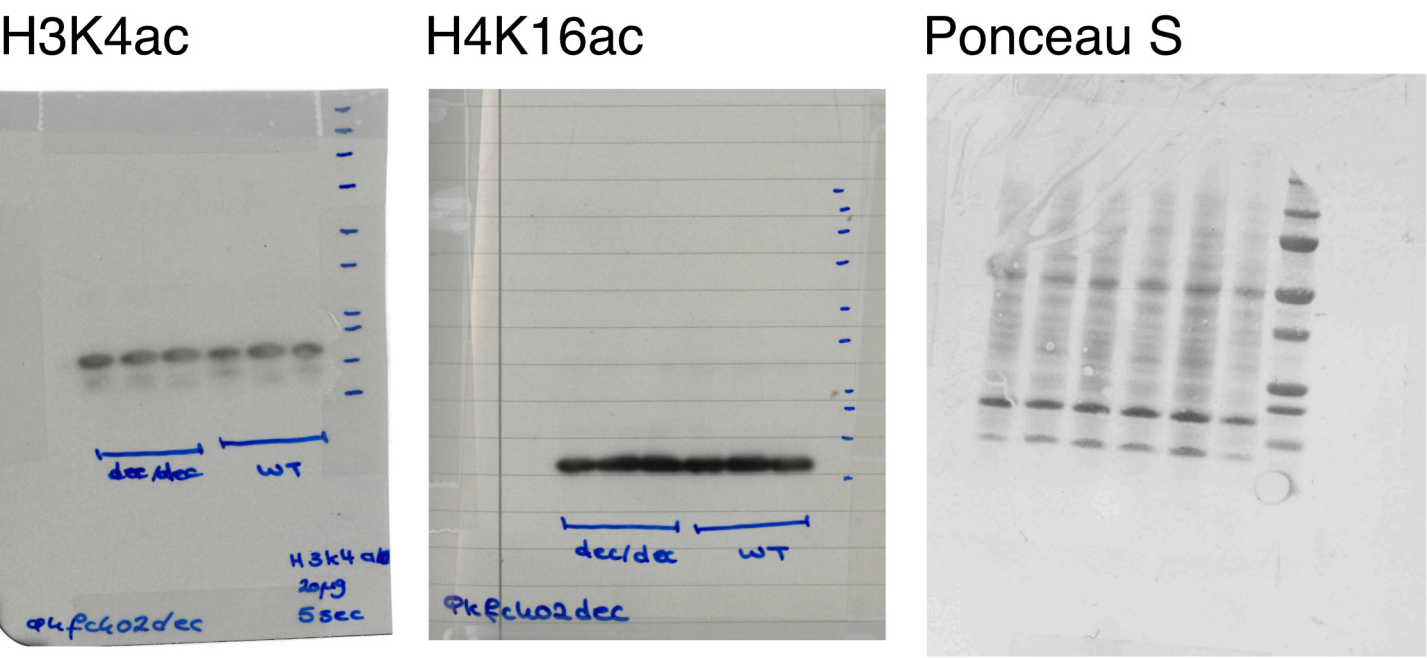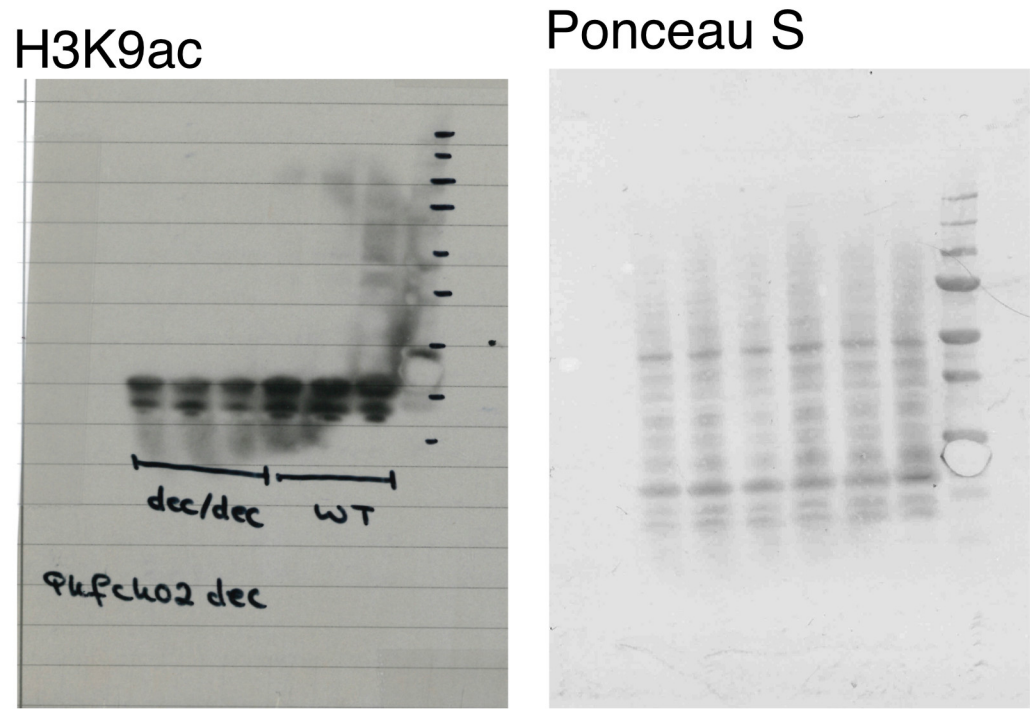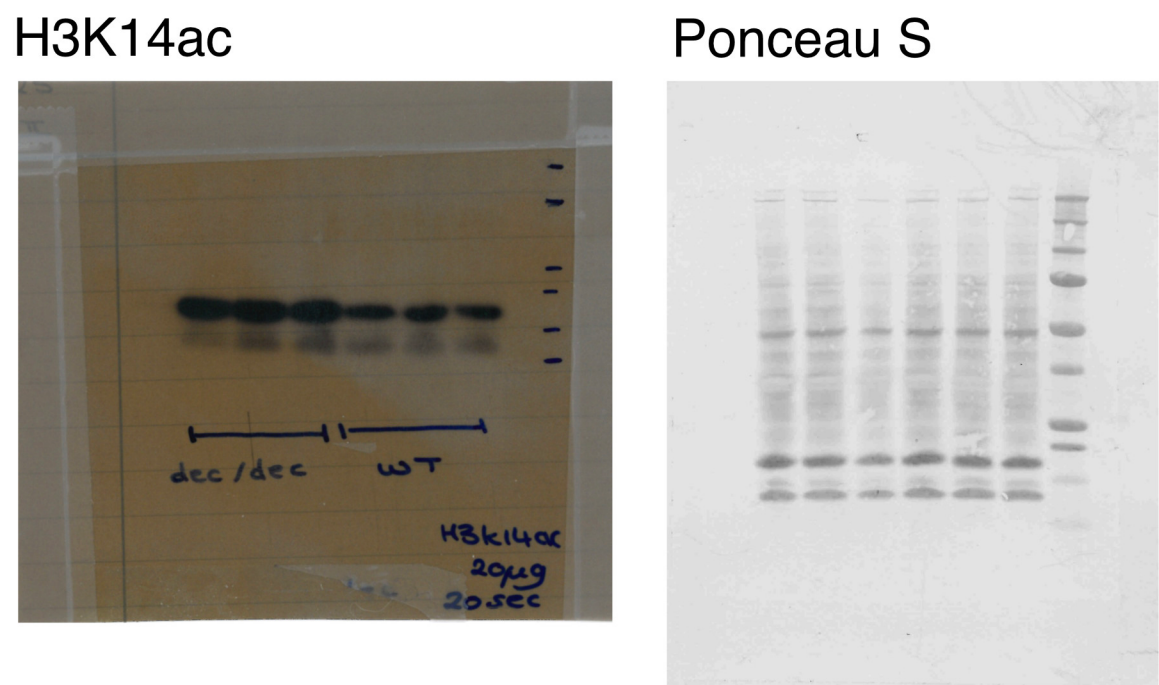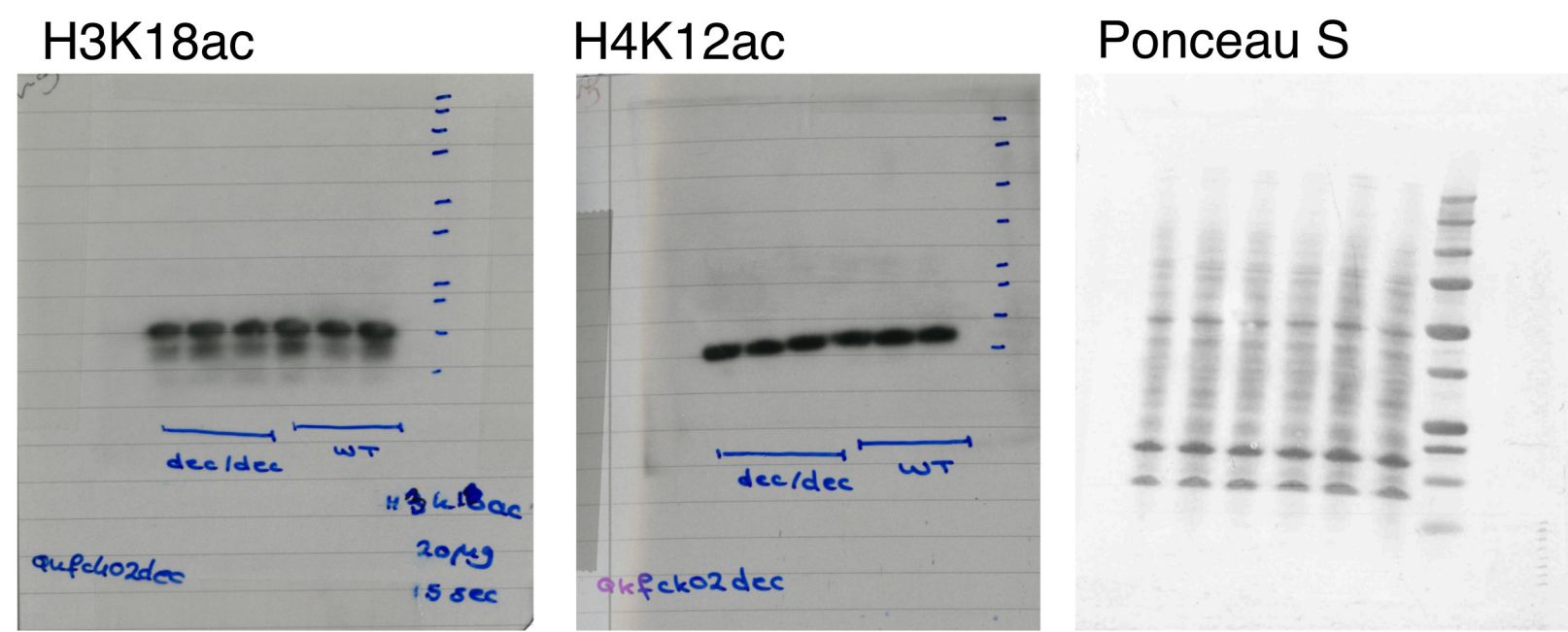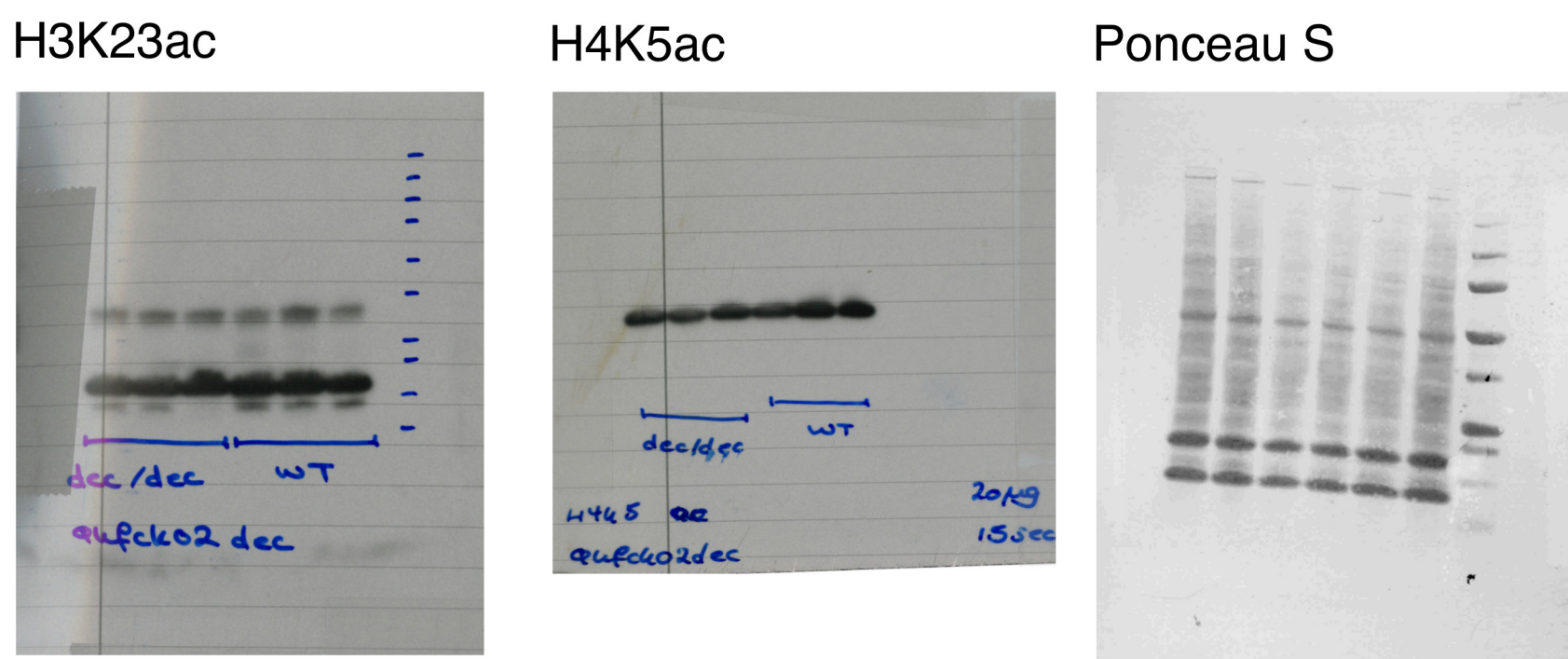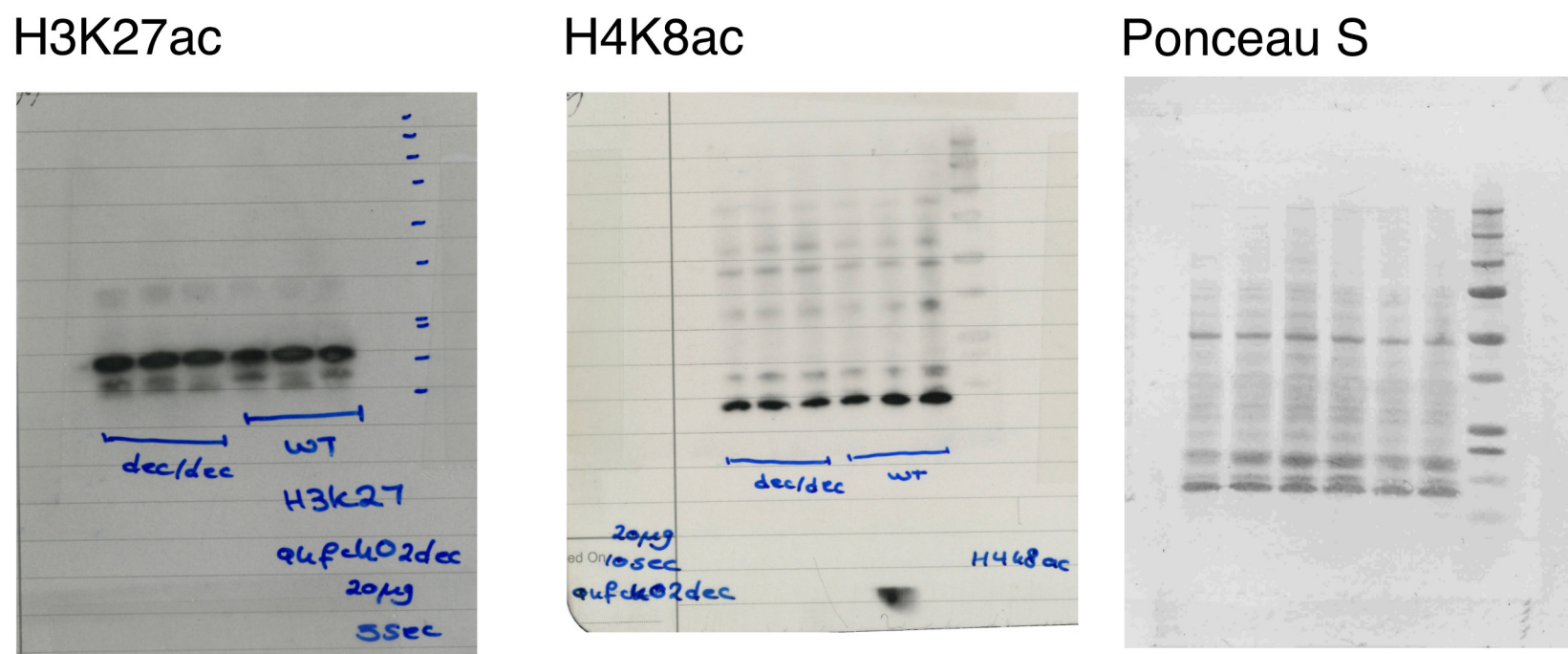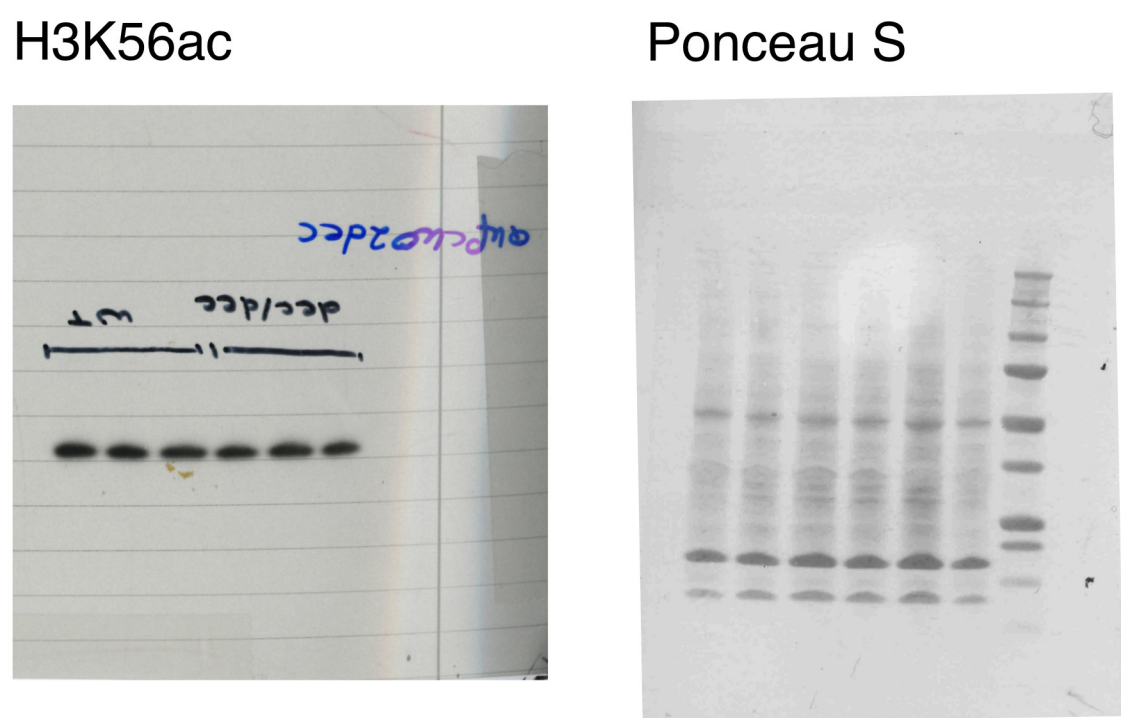

Supplemental Figure 2 Tg(Kat6b) vs. WT

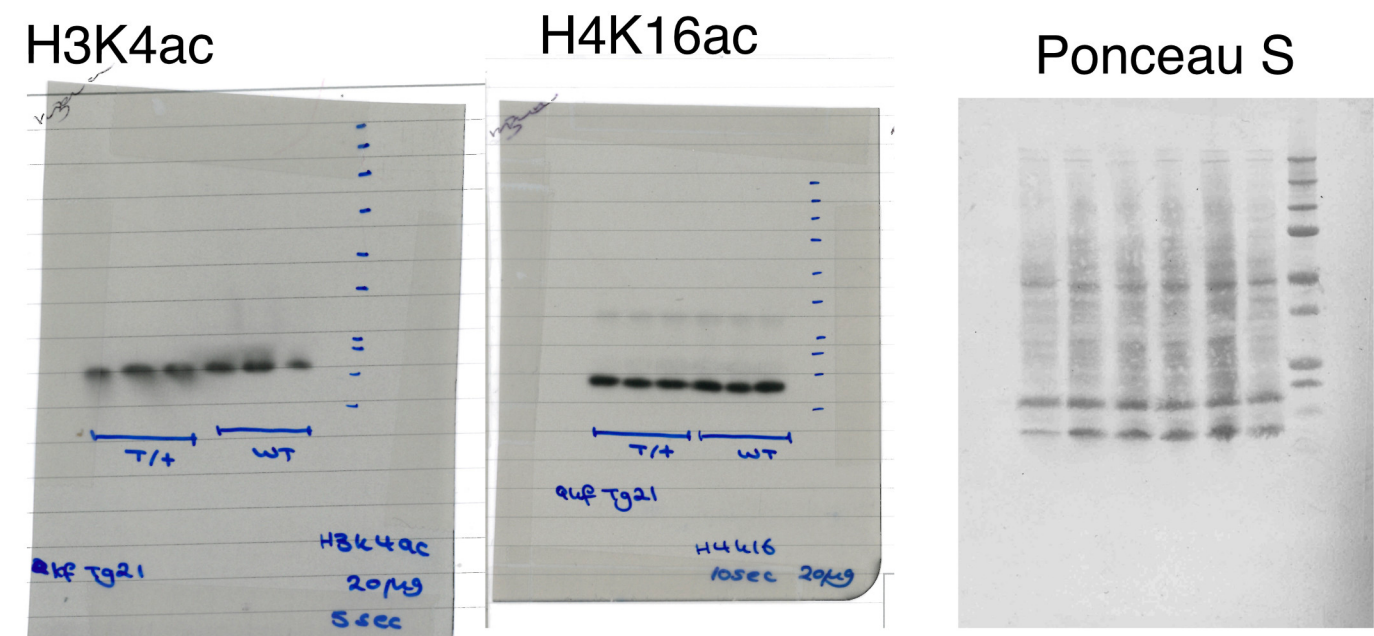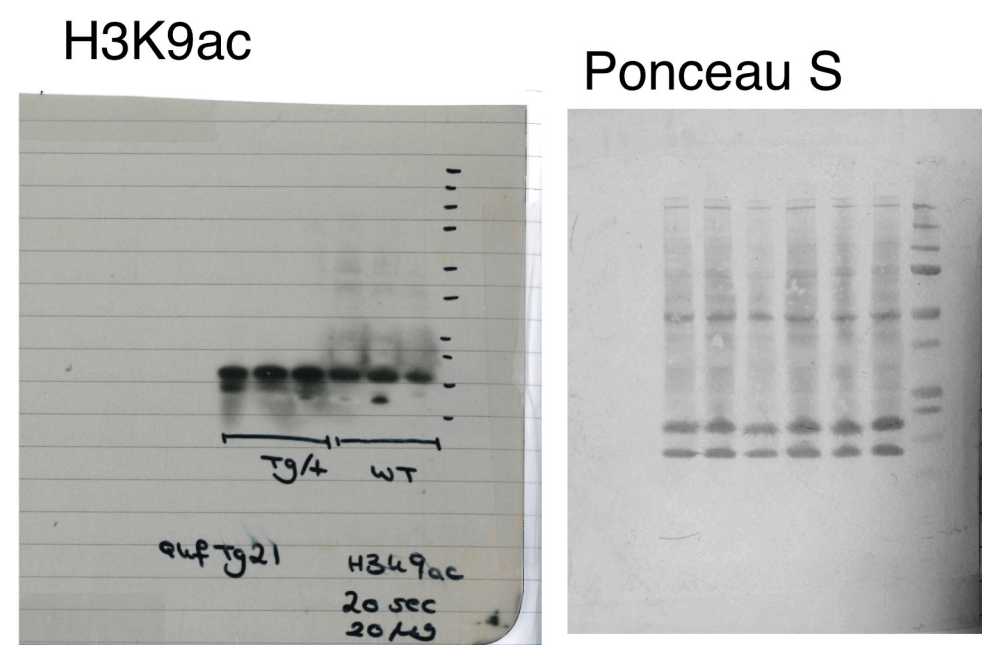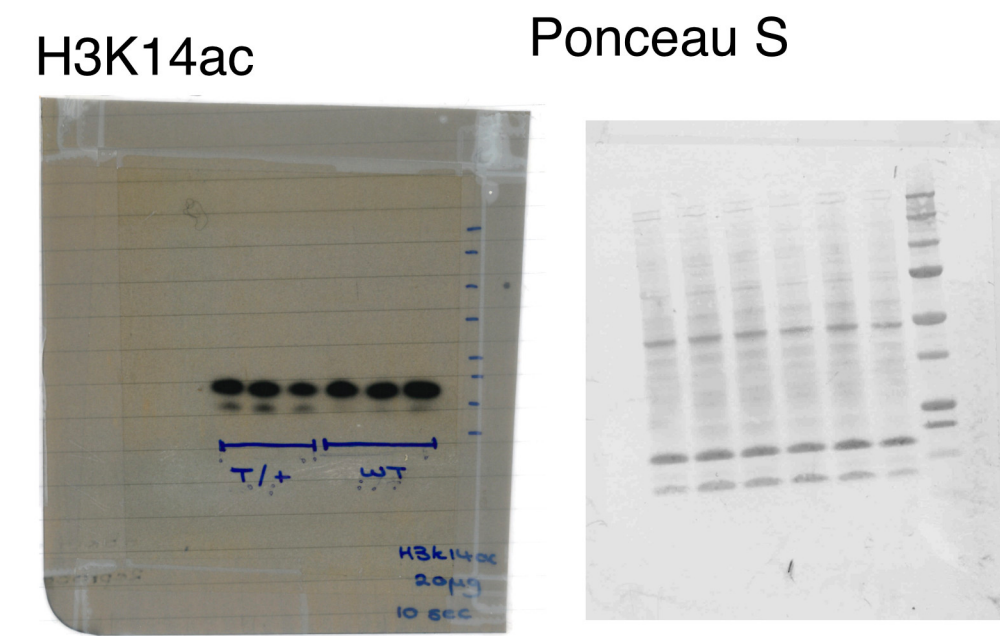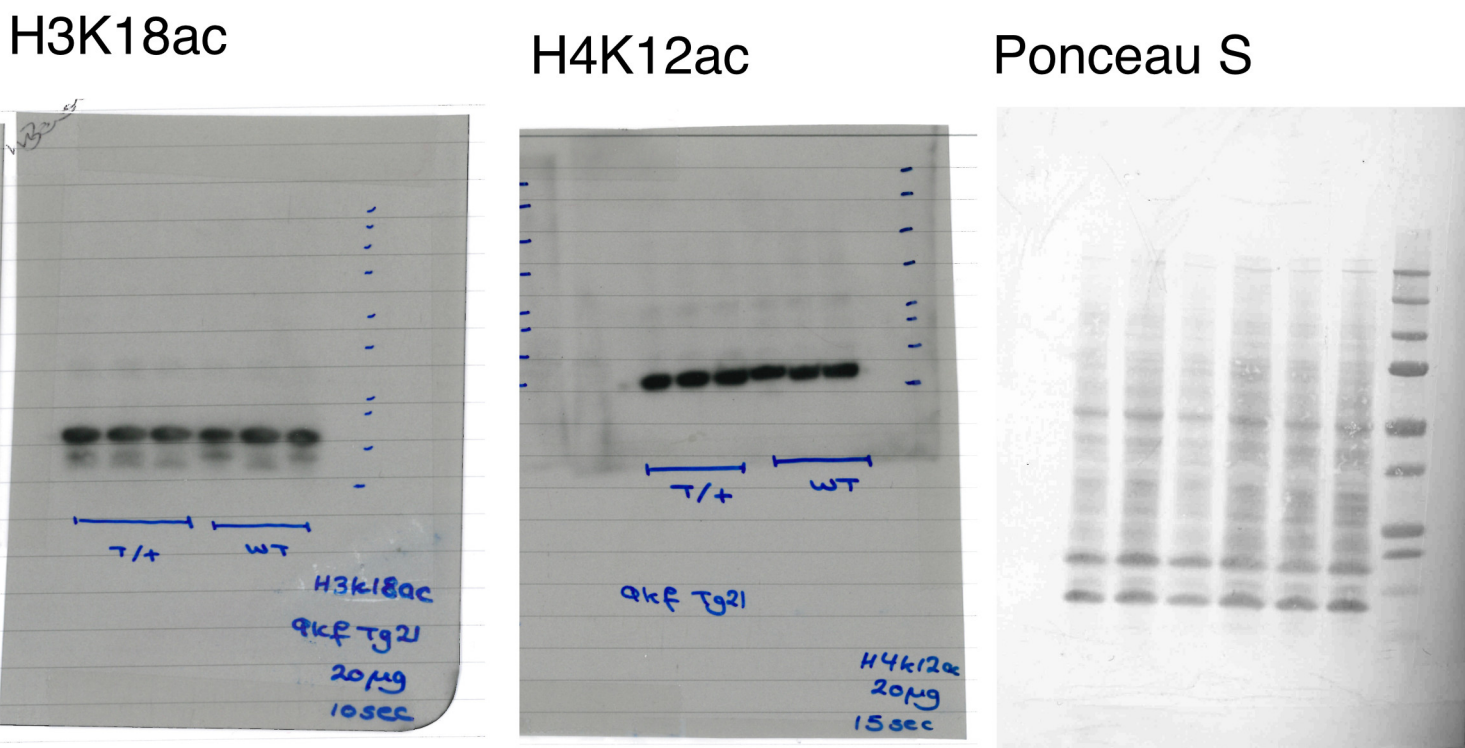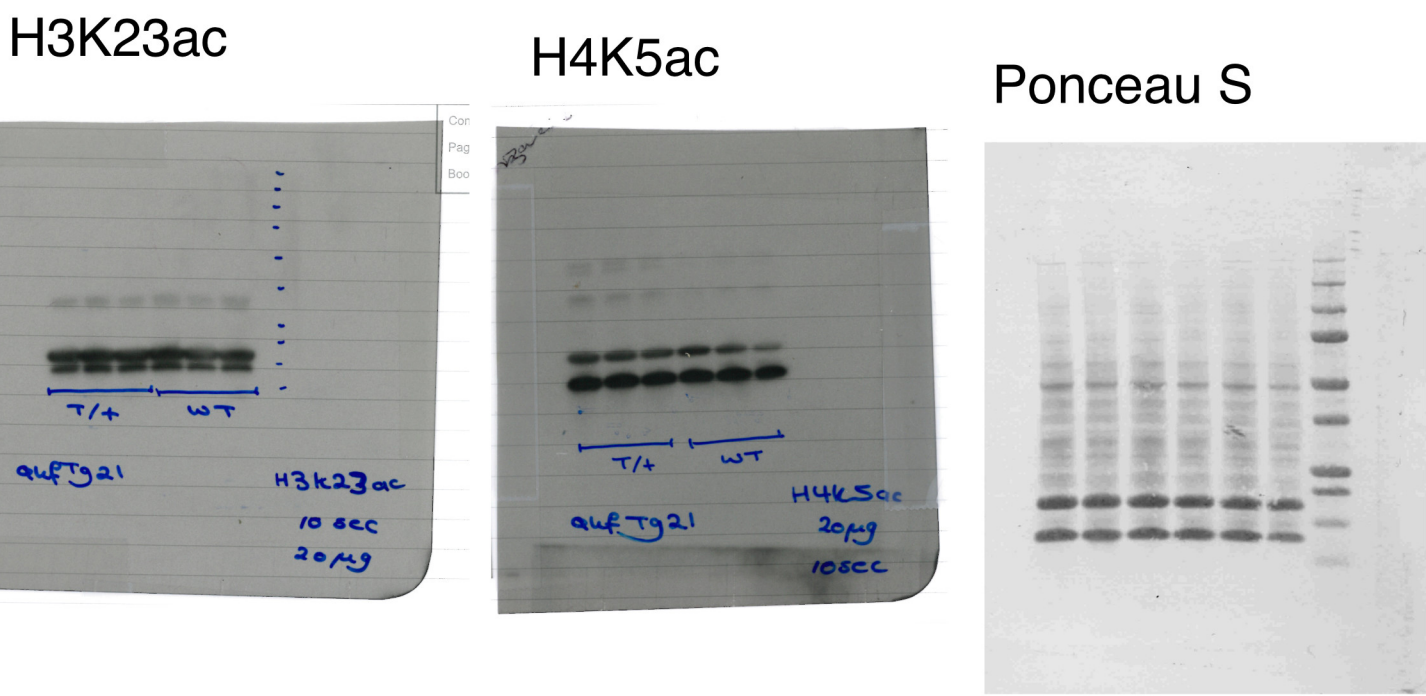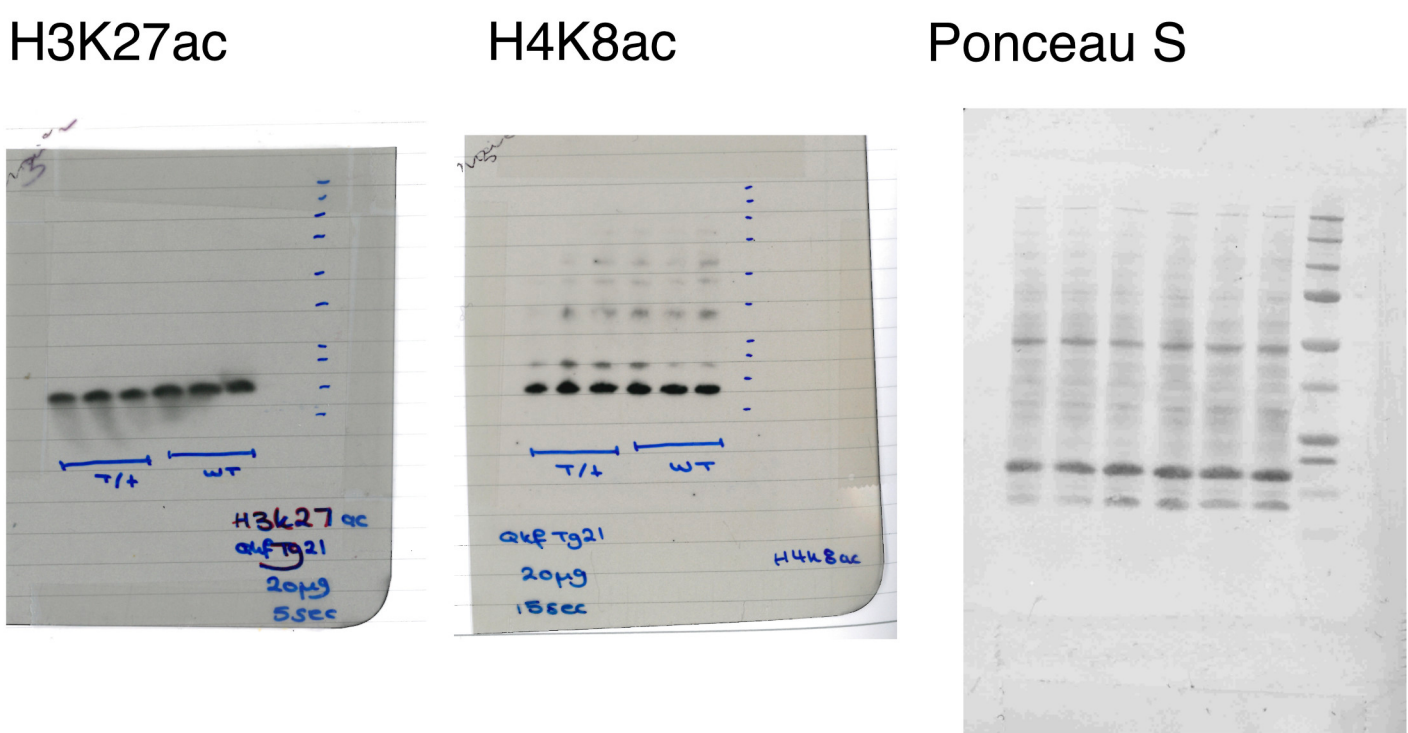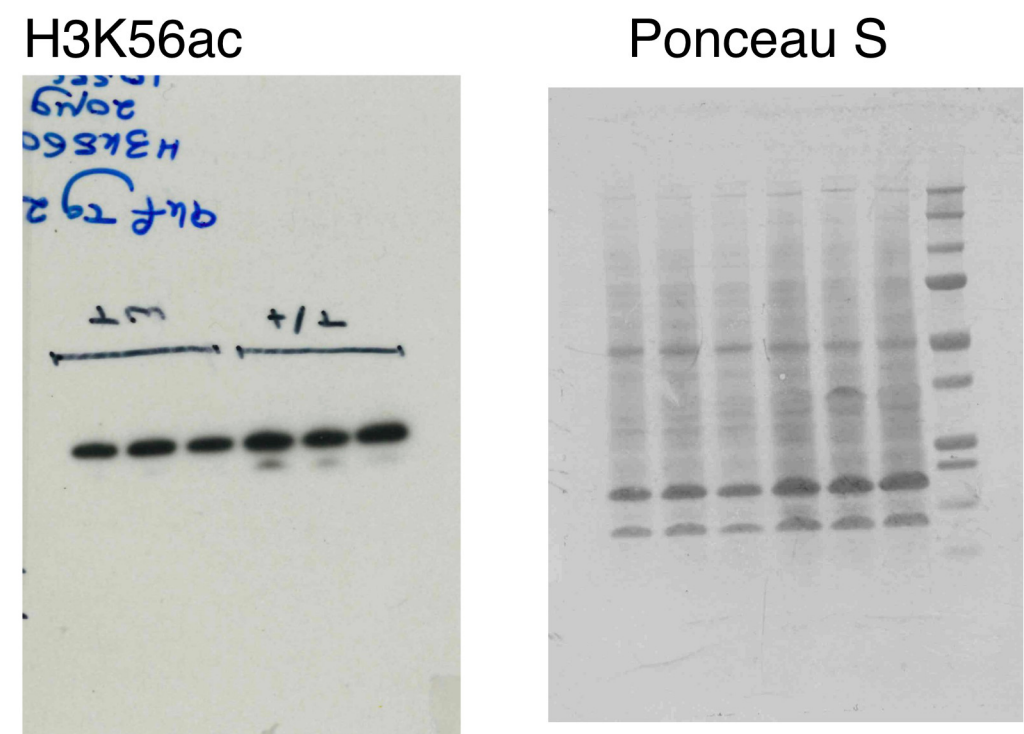

Supplement: Supplementary file 3 [file LSA-2024-02969_SdataFS2.pdf]

# Supplemental Figure 4 H3K14ac and panH3

NSPCs H3K14ac

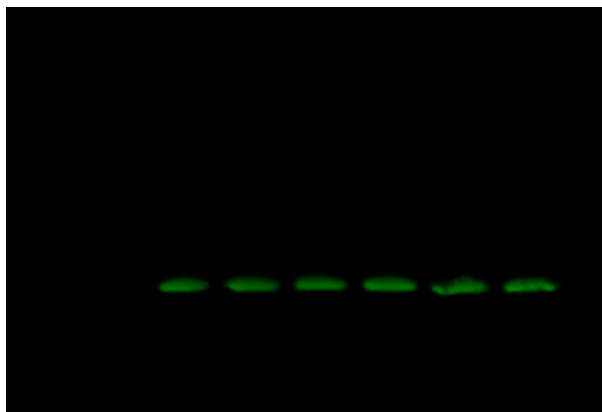

E12.5 dTel H3K14ac

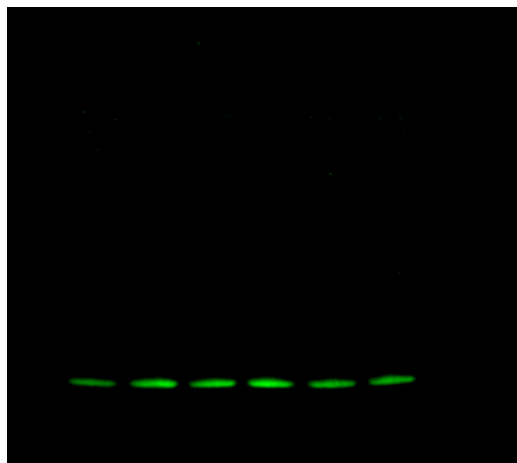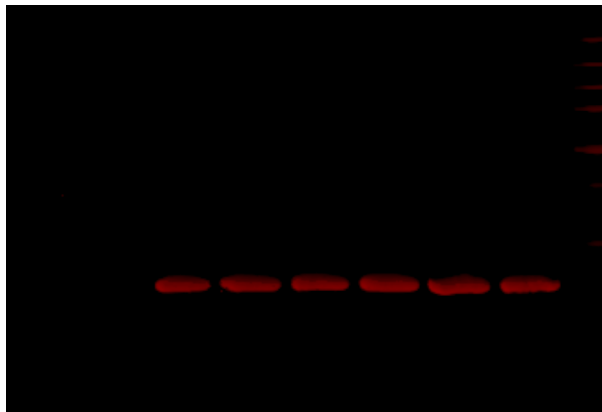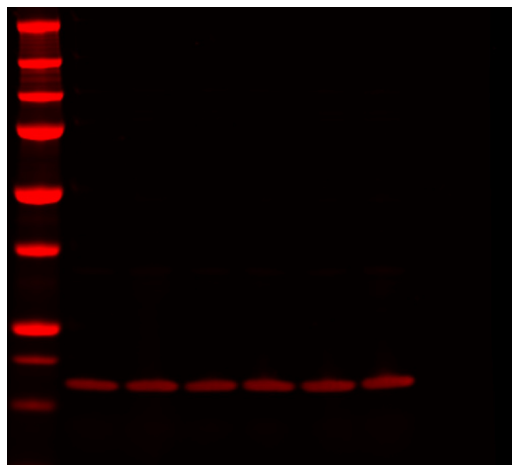

Supplement: Supplementary file 5 [file LSA-2024-02969_SdataFS4.pdf]
