# Supplementary material for: KAT6B is required for histone 3 lysine 9 acetylation and SOX gene expression in the developing brain
Source: Life Sci Alliance. 2024 Nov 13;8(2):e202402969. doi: 10.26508/lsa.202402969 (PMC11561263; doi:10.26508/lsa.202402969)
Supplement: Supplementary file 13 [file LSA-2024-02969_TableS5.docx]

**Supplemental Table 5: Genotyping primers**

| Targeted sequence | Primer sequence 5’ –> 3’ | PCR amplicons |
| --- | --- | --- |
| *Kat6b+*  *Kat6b –* | F1 TGCCTCAGAAAGCCATTACC  F2 CAGACAAATCAGCCCCAGAT  R GAGGCTCAGGGCTACAAGTG | WT = 230 bp  Del = 600 bp |
| *sacB (in BAC)* | F CAACTCAATCGACAGCTGGA  R GGCTTTGTTTGCCGTAATGT | sacB = 190 bp |
| *Kat6b-V5* | F CACGGCTACATGAACACAGG  R AGGCTTTCTTTCTGCTGCAT | WT = 229  V5 tag = 510 |
| *Sox2-GFP* | F AAGTTCATCTGCACCACCG  R TCCTTGAAGAAGATGGTGCG | Sox2-GFP = 173 bp |
