# Supplementary material for: KAT6B is required for histone 3 lysine 9 acetylation and SOX gene expression in the developing brain
Source: Life Sci Alliance. 2024 Nov 13;8(2):e202402969. doi: 10.26508/lsa.202402969 (PMC11561263; doi:10.26508/lsa.202402969)
Supplement: Supplementary file 14 [file LSA-2024-02969_TableS6.docx]

**Supplemental Table 6: Antibodies used for flow cytometry and immunofluorescence**

| Antibody | Catalogue number | Source |
| --- | --- | --- |
| Anti-SSEA1 | 347420 | BD Biosciences |
| Anti-CD133-APC | 17-1331-81 | eBioscience |
| Anti-mouse PE | N/A | In house |
| Anti-O4 AlexaFluor 594 | FAB1326T | R&D |
| Anti-βIII tubulin-PERCPCy5.5 | IC1195C | R&D |
| Anti-S100β | Ab52642 | Abcam |
| Anti-GFAP-AlexaFluor 488 | 53-9892-82 | Invitrogen |
| Anti-SOX2-APC | IC2018A | R&D |
| Anti-rabbit AlexaFluor 555 | A27039 | ThermoFisher |
| Fixable LIVE/DEAD^TM^ Violet Dead Cell Marker | L34964 | ThermoFisher |
| Anti-βIII tubulin | Promega | G7121 |
| Anti-GFAP | Dako | Z0334 |
| Anti-O4 AlexaFluor 488 | FAB1326G | R&D |
| Goat anti-mouse AlexaFluor 546 | A11003 | Invitrogen |
| Goat anti-rabbit AMCA | 711-155-152 | Jackson Immunoresearch |
