# Supplementary material for: KAT6B is required for histone 3 lysine 9 acetylation and SOX gene expression in the developing brain
Source: Life Sci Alliance. 2024 Nov 13;8(2):e202402969. doi: 10.26508/lsa.202402969 (PMC11561263; doi:10.26508/lsa.202402969)
Supplement: Supplementary file 15 [file LSA-2024-02969_TableS7.docx]

**Supplemental Table 7: Antibodies used for western blots, CUT&Tag and ChIP**

| Antibody | Concentration for western immunoblotting | Catalogue | Source |
| --- | --- | --- | --- |
| Anti-H3K4ac |  | 07-539 | Millipore |
| Anti-H3K9ac | 1:5000 | 9649 | Cell Signaling |
| Anti-H3K9ac | 1:5000 | 13001 | Epicypher |
| Anti-H3K14ac | 1:5000 | 7627 | Cell Signaling |
| Anti-H3K14ac | 1:1000 | Ab53946 | Abcam |
| Anti-H3K18ac | 1:2000 | Ab1191 | Abcam |
| Anti-H3K23ac | 1:5000 | 07-355 | Millipore |
| Anti-H3K27ac | 1:5000 | Ab4729 | Abcam |
| Anti-H3K56ac | 1:5000 | Ab76307 | Abcam |
| Anti-H4K5ac | 1:5000 | 07-327 | Millipore |
| Anti-H4K8ac | 1:5000 | 07-328 | Millipore |
| Anti-H4K12ac | 1:5000 | Ab46983 | Abcam |
| Anti-H4K16ac | 1:5000 | 07-329 | Millipore |
| RNA Polymerase II Subunit A (POLR2A), carboxy-terminal domain | not used in western blots | 05-623  clone CTD4H8 | Sigma |
| Guinea Pig anti-rabbit IgG | 1:100 | ABIN101961 | Antibodies online |
| Anti-mouse IRDye® 800 | 1:10000 | 926-32210 | Li-COR |
| Anti-Rabbit IRDye® 680 | 1:10000 | 926-68071 |  |
| Anti-rabbit HRP-conjugated IgG secondary | 1:10000 | NAV934v | Sigma |
