# Supplementary material for: KAT6B is required for histone 3 lysine 9 acetylation and SOX gene expression in the developing brain
Source: Life Sci Alliance. 2024 Nov 13;8(2):e202402969. doi: 10.26508/lsa.202402969 (PMC11561263; doi:10.26508/lsa.202402969)
Supplement: Supplementary file 16 [file LSA-2024-02969_TableS8.docx]

**Supplemental Table 8: Primers used for RT-qPCR**

| Targeted sequence | Primer sequence 5’ –> 3’ | Reference |
| --- | --- | --- |
| *Kat6b (mouse)* | F GTGCTTTTCCGTCCTCACTCC  R CACGATTTGACTCTTTAGTCCCC | Designed *de novo* |
| *Gapdh (mouse)* | F TGCACCACCAACTGCTTAGC  R GGCATGGACTGTGGTCATGAG | Wichmann et al., 2022 |
