# Supplementary material for: KAT6B is required for histone 3 lysine 9 acetylation and SOX gene expression in the developing brain
Source: Life Sci Alliance. 2024 Nov 13;8(2):e202402969. doi: 10.26508/lsa.202402969 (PMC11561263; doi:10.26508/lsa.202402969)
Supplement: Supplementary file 17 [file LSA-2024-02969_TableS9.docx]

**Supplemental Table 9: Primers used for Chromatin Immunoprecipitation qPCR**

| Targeted sequence | Primer sequence 5’-3’ | Distance from TSS | Reference |
| --- | --- | --- | --- |
| *Sox2* | F GAGCTTCTTTCCGTTGATGC  R TTCCCTACTCCACCAACCTG | -263 bp | Designed de novo |
| *Pax6* | F TTTAAACTCTGGGGCAGGTC  R GTGGGGTGTCAGGTGAGTCT | -100 bp | Designed de novo |

*TSS = Transcription start site
