# Supplementary material for: KAT6B is required for histone 3 lysine 9 acetylation and SOX gene expression in the developing brain
Source: Life Sci Alliance. 2024 Nov 13;8(2):e202402969. doi: 10.26508/lsa.202402969 (PMC11561263; doi:10.26508/lsa.202402969)
Supplement: Supplementary file 18 [file LSA-2024-02969_TableS10.docx]

**Supplemental Table 10: Nextera i5 and i7 indexing primers and generic p5 and p78 sequencing primers used in CUT&Tag and ATAC-sequencing (Mezger et al., 2018)**

| Name | Index sequence | Full sequence |
| --- | --- | --- |
| v2_P5.61 | CGATAGGG | AATGATACGGCGACCACCGAGATCTACACCGATAGGGTCGTCGGCAGCGTCAGATGTGTAT |
| v2_P7.60 | TTTAATGC | CAAGCAGAAGACGGCATACGAGATGCATTAAAGTCTCGTGGGCTCGGAGATGTG |
| v2_P7.63 | ATGATGAT | CAAGCAGAAGACGGCATACGAGATATCATCATGTCTCGTGGGCTCGGAGATGTG |
| v2_P7.65 | TAACAACA | CAAGCAGAAGACGGCATACGAGATTGTTGTTAGTCTCGTGGGCTCGGAGATGTG |
| v2_P7.67 | CATCGACC | CAAGCAGAAGACGGCATACGAGATGGTCGATGGTCTCGTGGGCTCGGAGATGTG |
| v2_P7.69 | CGGCCAAT | CAAGCAGAAGACGGCATACGAGATATTGGCCGGTCTCGTGGGCTCGGAGATGTG |
| v2_P7.78 | CGCCGTGC | CAAGCAGAAGACGGCATACGAGATGCACGGCGGTCTCGTGGGCTCGGAGATGTG |
| v2_P7.80 | CATTTCGA | CAAGCAGAAGACGGCATACGAGATTCGAAATGGTCTCGTGGGCTCGGAGATGTG |
| v2_P7.81 | GCTTGCCA | CAAGCAGAAGACGGCATACGAGATTGGCAAGCGTCTCGTGGGCTCGGAGATGTG |
| Illumina P5 | N/A | AATGATACGGCGACCACCGA |
| Illumina P7 | N/A | CAAGCAGAAGACGGCATACGAGAT |
